# Microbiomic and Metabolomic Analyses Unveil the Protective Effect of Saffron in a Mouse Colitis Model

**DOI:** 10.3390/cimb45070351

**Published:** 2023-06-30

**Authors:** Gulshan Singh, Hassan Brim, Yeneneh Haileselassie, Sudhir Varma, Aida Habtezion, Mudasir Rashid, Sidhartha R. Sinha, Hassan Ashktorab

**Affiliations:** 1 Division of Gastroenterology and Hepatology, School of Medicine, Stanford University, Palo Alto, CA 94305, USA; 2 Department of Pathology, Howard University College of Medicine, Washington, DC 20059, USA; 3 Hithru Analytics LLC, Silver Spring, MD 20877, USA; 4 Department of Pathology and Cancer Center, College of Medicine, Howard University College of Medicine, Washington, DC 20059, USA

**Keywords:** saffron, microbiome, metabolites, inflammation, colitis

## Abstract

Despite the existence of effective drugs used to treat inflammatory bowel disease (IBD), many patients fail to respond or lose response over time. Further, many drugs can carry serious adverse effects, including increased risk of infections and malignancies. Saffron (*Crocus sativus*) has been reported to have anti-inflammatory properties. Its protective role in IBD and how the microbiome and metabolome play a role has not been explored extensively. We aimed to establish whether saffron treatment modulates the host microbiome and metabolic profile in experimental colitis. Colitis was induced in C57BL/6 mice with 3% DSS and treated with either saffron in a dose of 20 mg/kg body weight or vehicle through daily gavage. On day 10, stool pellets from mice were collected and analyzed to assess saffron’s effect on fecal microbiota and metabolites through 16S rRNA sequencing and untargeted primary metabolite analysis. Saffron treatment maintained gut microbiota homeostasis by counter-selecting pro-inflammatory bacteria and maintained Firmicutes/Bacteroides ratio, which was otherwise disturbed by DSS treatment. Several metabolites (uric acid, cholesterol, 2 hydroxyglutaric acid, allantoic acid, 2 hydroxyhexanoic acid) were altered significantly with saffron treatment in DSS-treated mice, and this might play a role in mediating saffron’s colitis-mitigating effects. These data demonstrate saffron’s therapeutic potential, and its protective role is modulated by gut microbiota, potentially acting through changes in metabolites.

## 1. Introduction

Inflammatory bowel disease (IBD) is a chronic inflammatory disorder of the gut. Two major defined forms of IBD include ulcerative colitis (UC) and Crohn’s disease (CD). IBD has become a global disease. Its incidence has persistently increased, with the disease now affecting over 6.8 million people [1,2,3]. There are unmet needs with respect to new and safe therapies since many patients lose response to anti-inflammatory and immunosuppressive drugs over time [4,5]. Further, there are many serious adverse risks associated with many of the current therapies [6]. There has been a lot of research conducted to help decode the causes of IBD, such as the genetic interaction between the human gut microbiome and the mucosal immune system and alterations in environmental factors [7]. The use of natural products to develop diagnostic and therapeutic strategies is increasingly being tested in IBD patients. The use of herbal compounds among patients with ulcerative colitis is well-known in the western world and in many Asian countries [8]. There are several natural products that have been under investigation for the treatment of IBD [9], such as Aloe vera [10], wheat grass (*Triticum aestivum*) [11], Curcumin [12,13], and Licorice [14], but controlled data indicating the efficacy of these alternative herbal agents is limited in the management of patients with UC and milder diseases [15].

The structure and the composition of the gut microbiota is severely disturbed in IBD patients compared to healthy individuals [16,17,18]. The imbalances of the Firmicutes and Bacteroidetes at the phylum level and other bacterial families such as Ruminococcaceae, Veillonellaceae, Christensenellaceae, Bacteroidaceae, and Rikenellaceae have been reported in IBD patients [18,19,20]. In addition to disrupted microbial profile, IBD patients have an altered metabolic profile [17].

Metabolites are small molecules which act as bridging components between the intestinal microbiome and the host immune system [21]. Some of these metabolites rely on gut microbiota for their production. Our group recently showed dysbiosis associated with a deficiency in bacterial metabolites (secondary bile acids, SBAs) in UC patients with pouches compared to control familial adenomatous polyposis (FAP) subjects with pouches [20]. Moreover, in this study, treatment with localized SBAs mitigated inflammation in multiple experimental models of colitis/IBD.

*Crocus sativus* L. (Iridaceae), commonly known as saffron, is cultivated worldwide, especially in Iran, India, Greece, Morocco, Spain, Italy, and China. Saffron has several biological activities and is used in traditional medicine [22]. The constituents of saffron (picocrocin, crocetin, and its glycoside crocins) have been found to have various therapeutic effects, such as anti-depressant, anti-inflammatory, and anti-cancer activities [23,24,25]. We have recently reported the protective effect of saffron extracts in mouse colitis models through immune modulation [26]. Dietary constituents are known to impact the microbiota [27]. However, little is known regarding the effects of saffron on the gut microbiome. Exploring the therapeutic role of saffron in an experimental model will help to delineate possible mechanisms for its protective capabilities by better understanding the effects on microbial and metabolic profiles associated with intestinal inflammation. 

## 2. Materials and Methods

### 2.1. Animals

C57BL/6 were purchased from Taconic (Hudson, NY, USA) and housed at the Stanford research animal facility for 1 week until used in experiments. Animals were maintained in accordance with the National Institutes of Health guidelines, and experiments were approved by Stanford University Institutional Animal Care and Use Committee the APLAC-24685. We used 4 mice per group in this study since our previous studies in rodents have shown the reproducible effect of saffron [23,26,28].

### 2.2. Saffron Aqueous Extract

Dried saffron stigmas were obtained from Gulf Pearls SPRL (Brussels, Belgium, www.gp-food.com, accessed on 7 June 2023). The quality of saffron was evaluated for batch-to-batch variation according to ISO3632 standard and our previous study [29]. Dried saffron stigmas were ground into fine powder and dissolved in molecular grade distilled water at a concentration of 2 mg/mL in a 15 mL falcon tube after filtration using 0.45 μM filter on an orbital shaker at room temperature in the dark for one hour. This is called saffron suspension/aqueous extract (SFE). The SFE can be stored for two days at 4 °C. The extraction was prepared fresh every second day for oral administration by gavage. The stability of saffron extracts was analyzed as per our previous study.

### 2.3. Dextran Sodium Sulfate (DSS) Colitis Model

**Figure 1:**
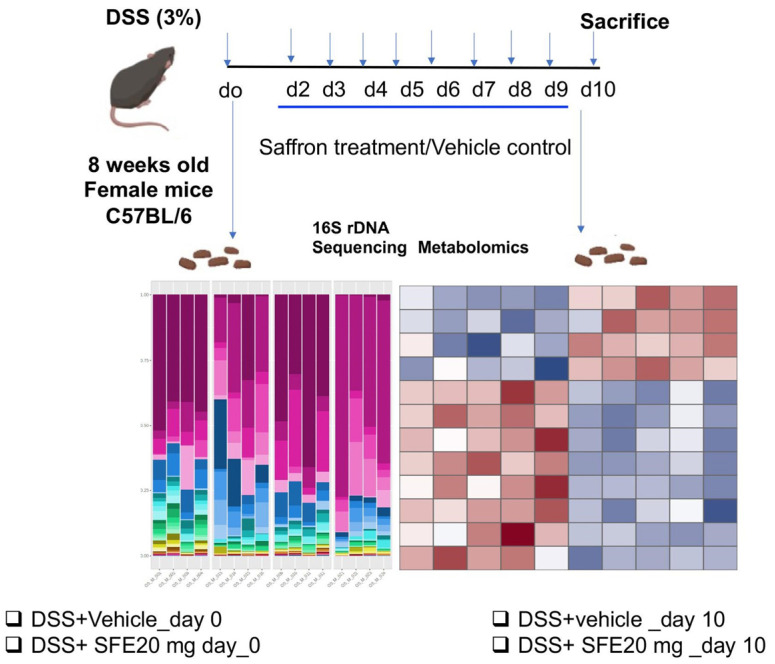
Schematic diagram representing methodology of the experiment of DSS colitis model and collection of stool samples for metabolomics and microbiomics.

DSS (36,000–50,000 MW) was purchased from MP Biomedicals (Santa Ana, CA, USA) and dissolved in drinking water to 3% (*w*/*v*) and given ad libitum to 8-week-old female C57BL/6 mice beginning on day 0 for 10 days. In the control group, the mice were given regular drinking water. We induced colitis via administration of 3% dextran sodium sulfate (DSS) in drinking water in wild type C57BL/6 mice (day 0–10) and, at day 2, mice were gavaged daily with either saffron aqueous extract (SFE) or vehicle control until day 10. The effective dose of saffron, i.e., the dose that has a therapeutic effect, has already been established by our group [26]. The most effective dose of saffron, 20 mg/kg b.wt, was used for microbiome and metabolomics experiments. On day 0 and 10, stool pellets were collected from the mice by keeping them in individual cages and immediately snap frozen and stored in −80 before shipping samples to the cores for bacterial 16S rRNA gene sequencing and untargeted primary metabolites analyses (Figure 1, supplementary Figure S1).

### 2.4. Microbiome Analysis

The Genome Sequencing Service Center (GSSC) at Stanford University conducted DNA sequencing [20]. Details of OTU assembly from reads, abundance estimation of each out, and taxonomic assignment are as reported in previous studies [23,30]. OTU abundances were loaded into R (version 3.5.1) using the phyloseq package [31]. To determine whether the sequencing depth was adequate to fairly represent the OTU richness of the samples, we performed a rarefaction analysis. To check how the samples from the same experimental condition cluster with each other, we generated a Principal Coordinate Analysis plot using the Jensen–Shannon Divergence distance measure. Indeed, the day 10 samples in DSS treatment clustered together and distinctly from the day 10 samples from DSS + SFE 20 mg/kg b.wt mice. This finding led us to pool samples from the mice in each treatment group for more specific analyses at the phylum, order, and genus levels to establish saffron-induced differentials at the gut microbiota composition level.

To detect taxonomic units that are differentially expressed between Saffron-treated and DSS group, we performed two-factor ANOVA, with treatment (saffron vs. untreated) and days (0 and 10) as the two factors. The analysis was carried out by using the R package DESeq2 [32]. At each taxonomic rank, we summed up the counts of reads falling into OTUs within each taxon. Those counts were then used to perform the ANOVA using DESeq2. Taxons with a false detection ratio (FDR) < 0.05 were selected as statistically significant. In addition, we used microbiome Analyst software for some microbiome analysis. A minimum count of 4 and a 20% prevalence filter were used to filter low-quality reads. For community-level comparisons, such as estimation of beta diversity, Total sum scaling normalization (TSS) was performed. Regarding DSS treatment (in this study for 10 days), it was established that the alpha diversity tends to decrease [33,34]. In both groups (DSS + Vehicle and DSS + SFE20), after treatment with DSS (either with vehicle or Saffron), there was a decrease in alpha diversity, which shows that, with DSS treatment, there is a decrease in alpha diversity index irrespective of Saffron intervention.

### 2.5. Untargeted Metabolomics

Untargeted metabolomic analysis of the frozen stool samples was performed via the NIH-supported Michigan Regional Comprehensive Metabolomics Resource Core (MRC2). The methodology of untargeted metabolomics has been previously published [35]. Briefly, the protein precipitation of the weight-adjusted stool was initiated with the addition of 600 µL MAA containing 2 µM recovery standard (IS). The samples were sonicated to homogenize the samples. Following centrifugation, 200 µL supernatant of the samples were transferred into new tubes and brought to complete dryness under the continuous stream of N_2_. Metabolites separated by positive and negative ions were analyzed using LC-MS (Liquid chromatography-mass spectrometry). Raw data were processed using Agilent MassHunter Qual (v. B.08.00) and Profinder (v. B.08.00) software. The peak intensities obtained from the mass spectrometry were further analyzed using MetaboAnalyst [36]. Primary metabolism was performed as follows: Samples were extracted using 1 mL of 3:3:2 can(acetonitrile): IPA(Iso-propyl alcohol): H2O (*v*/*v*/*v*). Half of the samples were dried to completeness and then derivatized using 10 μL of 40 mg/mL of Methoxyamine in pyridine before being shaken at 30 °C for 1.5 h. Then, 91 μL of MSTFA (N-Methyl-N-(Trimethylsilyl)trifluoroacetamide + FAMEs (fatty acid methyl esters) was applied to each sample before they were shaken at 37 °C for 0.5 h to finish derivatization. Samples were then vialed, capped, and injected onto the instrument. We used a 7890A GC coupled with a LECO TOF. A total of 0.5 μL of derivatized sample was injected using a splitless method onto a RESTEK RTX-5SIL MS column with an Intergra-Guard at 275 °C with a helium flow of 1 mL/min. The GC oven was set to hold at 50 °C for 1 min then ramp up to 20 °C/min to 330 °C and then hold for 5 min. The transferline was set to 280 °C while the EI ion source was set to 250 °C. The Mass spec parameters collected data from 85 *m*/*z* to 500 *m*/*z* at an acquisition rate of 17 spectra/s.

### 2.6. Statistical Analysis

Statistical analyses were performed with Prism 7 (GraphPad Software, Inc., La Jolla, CA, USA). Differences with *p* < 0.05 were regarded as statistically significant. For estimating alpha diversity, the Wilcoxon rank-sum test was used to assess differences in richness and Shannon index. For estimating β-diversity, Jaccard and Bray–Curtis index were used and visualized using either PCoA or nonmetric multidimensional scaling (NMDS). Ordination between the groups was assessed for statistical significance using Permutation Multifactorial Analysis of Variance (PERMANOVA). For metabolite comparisons, one-way ANOVA with Tukey’s post hoc correction was performed on the log-transformed values. Student’s *t*-test was performed to compare the two groups.

## 3. Results

### 3.1. Saffron Treatment Alters DSS-Induced Microbial Profile

**Figure 2:**
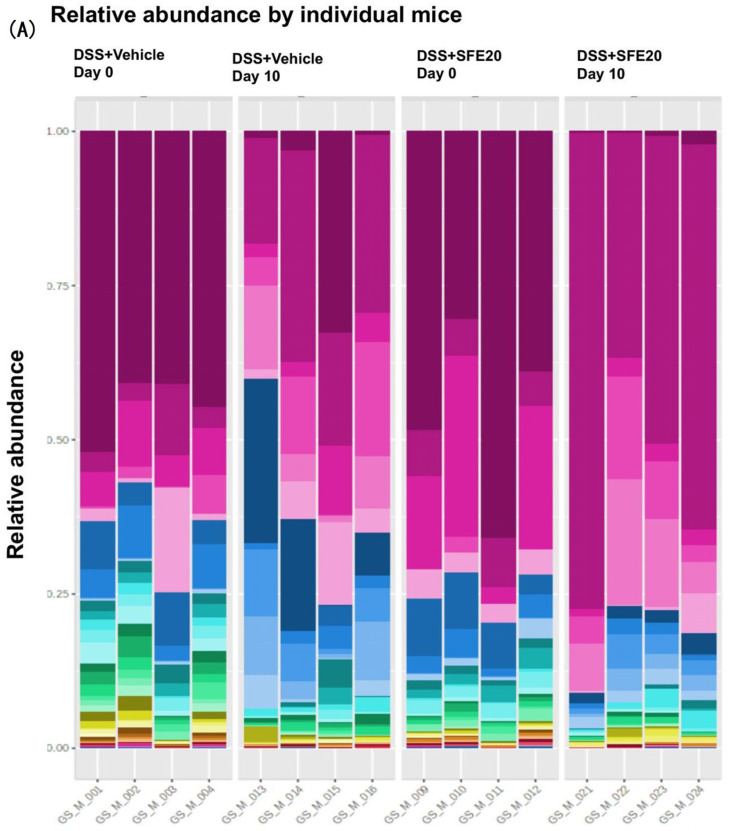

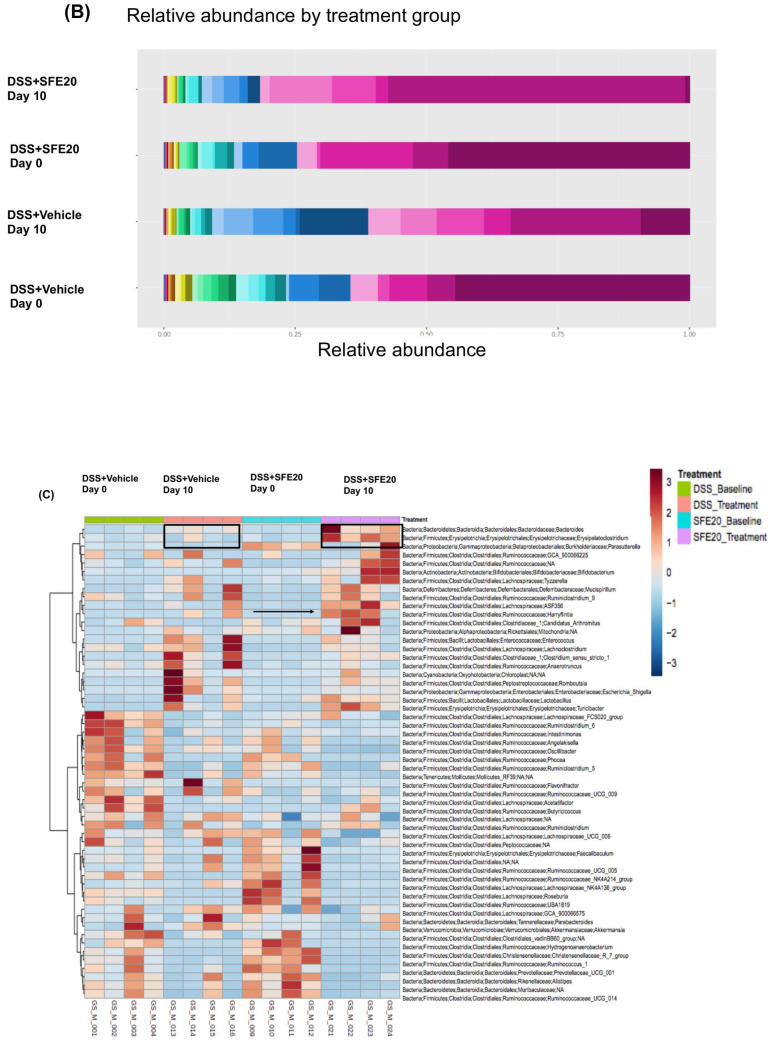
Representation of microbiome relative abundance at two time points (day 0 and day 10) between DSS + vehicle and DSS + SFE20 mg groups. (**A**) Relative percent bacterial abundance of individual subject at phylum level, (**B**) relative percent bacterial abundance of treatment week at phylum level, (**C**) hierarchical clustering and heatmap showing saffron treatment changes DSS-induced microbiome profile.

To determine the effects of saffron on the composition of the gut microbiota in the mice treated with DSS, a high-throughput sequencing of 16 S rRNA was performed. We assessed the effect of saffron on the microbial population by comparing DSS + vehicle group and DSS + SFE 20 mg/kg b.wt group at day 0 and 10. The predominant phylum/families in the stool samples in mice were Bifidobacterium, Bacteroides, Muribaculaceae, Prevotellaceae_UCG_001, Alistipes, Parabacteroides, Cholorplast, Mucispirillum, Enterococcus, Lactobacillus (Figure 2A,B, supplementary Figure S2). A heatmap visualization of the relative abundance of the microbiota in stool samples from mice treated with DSS and saffron/vehicle at day 0 and day 10 showed that saffron treatment changes the DSS-induced microbiome profile (Figure 2C).

**Figure 3:**
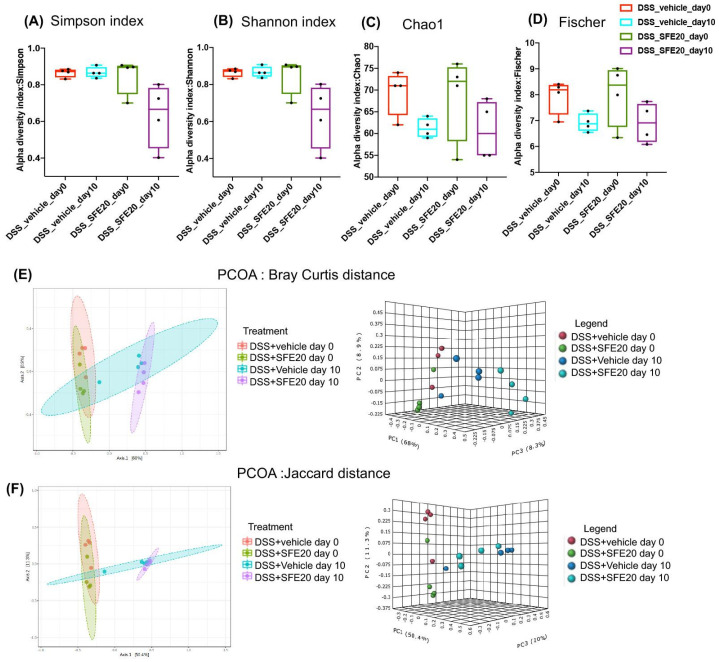
Microbial diversity comparison between two timepoints (day 0 and day 10) between DSS + vehicle and DSS + SFE20 mg groups. The OTU number representing the bacterial species richness of the microbiota was estimated for alpha diversity using the following: (**A**) Simpson (*p* = 0.018), (**B**) Shannon index (*p* = 0.007), (**C**) Chao1 (*p* = 0.172), (**D**) Fischer (*p* = 0.126). (**E**) β-diversity using PCOA (Bray–Curtis, [PERMANOVA] F-value: 11.132; R-squared: 0.736; *p*-value < 0.001, (**F**) Jaccard distance PERMANOVA] F-value: 6.4871; R-squared: 0.618; *p*-value < 0.001.

Alpha diversity in DSS-treated mice with or without saffron intervention at two time points (day 0 and day 10) was significantly altered, as revealed by Simpson (*p* = 0.018), Shannon index (*p* = 0.007), Chao1 (*p* = 0.172), Fischer (*p* = 0.126) between saffron-treated and vehicle group at day 0 and day 10 (Figure 3A–D). 

β-diversity using PCOA (Bray–Curtis, [PERMANOVA] F-value: 11.132; R-squared: 0.736; *p*-value < 0.001, F), Jaccard distance PERMANOVA] F-value: 6.4871; R-squared: 0.618; *p*-value < 0.001 revealed a shift in microbial diversity at two time points (day 0 day 10) and in DSS-treated mice with or without saffron intervention when compared at day 10 (Figure 3E,F). 

### 3.2. Saffron Alters the Abundance of Microbial Taxa Associated with Inflammation

**Figure 4:**
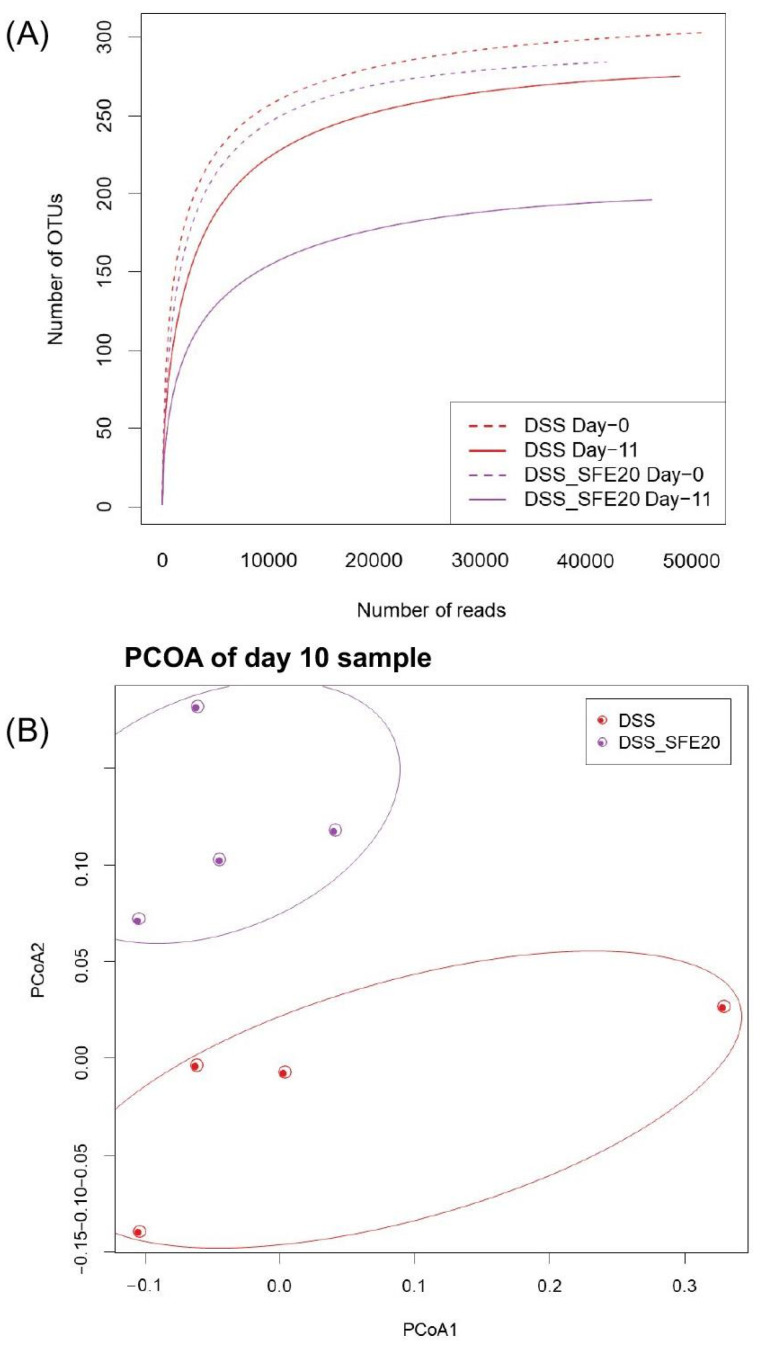

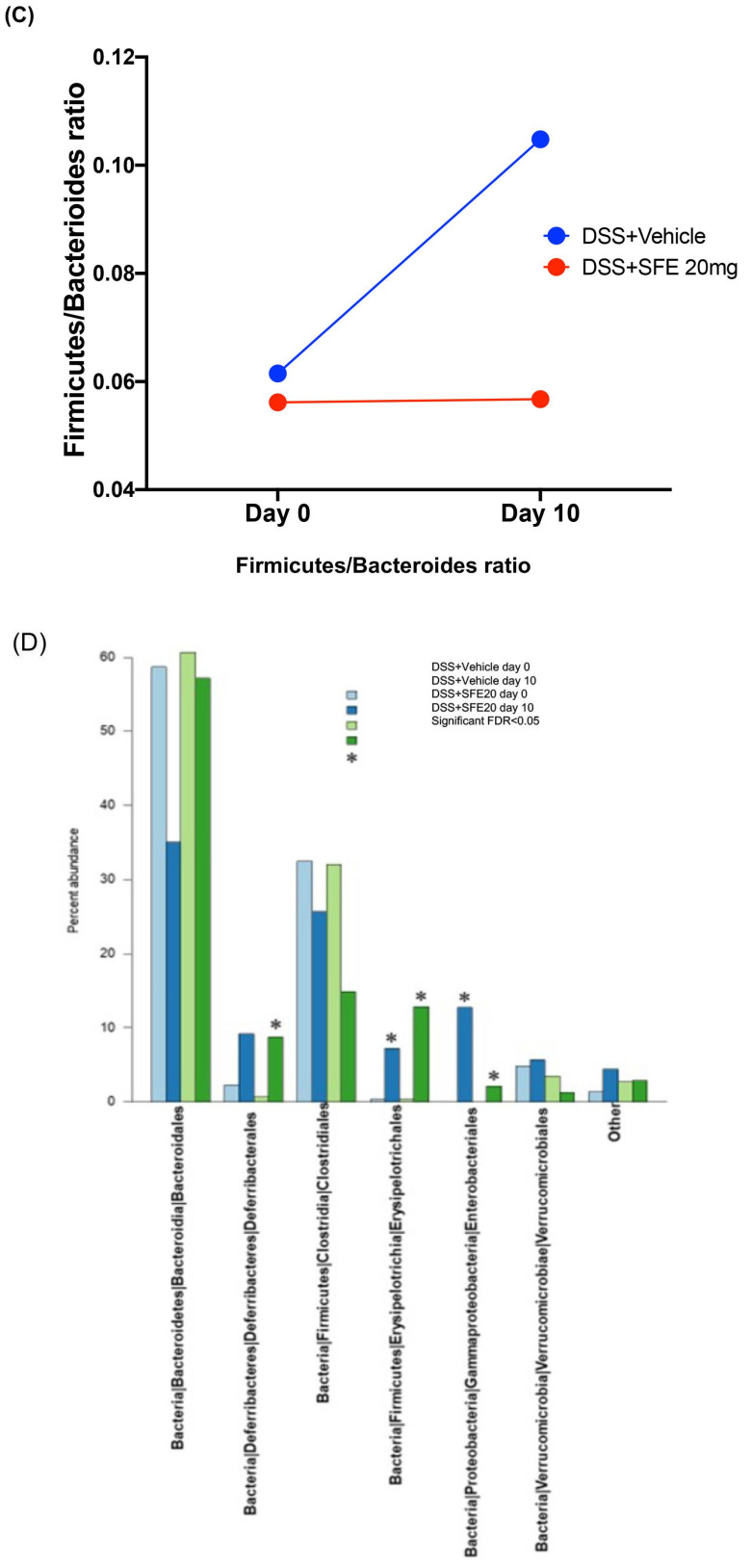

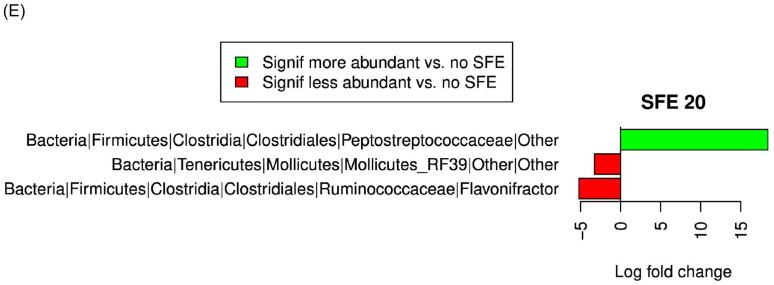
Saffron increases the abundance of gut microbial taxa associated with maintenance of microbiome homeostasis (**A**). Firmicutes/Bacteroidetes ratio in DSS (day 0, 579 and day 10, 0.974) and DSS-SFE (day 0, 0.548, day 10, 0.501) show maintenance of microbiome hemostasis in saffron-treated animals (**B**). Relative abundance of bacterial order in the colonic mucosa (**C**). Bars represent mice group. (**D**) Labels indicate families with average relative abundances >1% in at least one treatment group (**E**). Relative abundance of mucosa-associated bacteria at genus was significantly altered in saffron-treated animals. Data are presented as means ± SEM. * *p* < 0.05 DSS vs. DSS + SFE gavaged mice.

Rarefaction and Shannon index analysis indicated that the sequencing depth covered the major and minor phylotypes and that most of the diversity was accounted for (Figure 4A, Supplementary Table S1). The average rarefaction curves for each experimental condition reveal that all the curves are close to saturation, indicating that the sequencing depth is adequate for the detection of all OTUs in the analyzed samples. Moreover, the saturation curve (curve flattening) at day 10 under saffron treatment was reached earlier than in the other experimental samples and at a lower number of sequencing reads. This probably attests to the higher richness among OTUs, attributable to saffron treatment for 10 days. However, overall, in post hoc analysis, the diversity decreased in the treated group, but there is an insufficient number of samples to deem this to be statistically significant (Supplementary Table S1).

To further appreciate saffron-associated effects at the microbiota level, we performed a principal coordinate analysis (PCoA) of the DSS and DSS + SFE samples using UniFrac-based principal coordinate analysis (Figure 4B). Color-coded data from individual mice formed distinct components in this analysis. The ovals represented clustering by DSS treatment groups and were switched to those in the group along the PC1 axis in the saffron-treated group after the administration of saffron (Figure 4B). This analysis further highlighted the effect of saffron on gut microbiota composition, even in the in the presence of inflammation induced by DSS. To define the nature of saffron’s effects on the microbiota, in this colitis model, we assessed its impact on an important parameter in microbiome homeostasis, that is, the Firmicutes/Bacteroides ratio. This ratio was maintained between day 0 (0.579) and day 10 (0.501) in the DSS-treated mice gavaged with saffron. However, mice treated with DSS only had a high ratio at day 10 (0.974) compared to day 0 (0.579), attesting to an enrichment of Firmicutes and/or a depletion of Bacteroides under DSS treatment (Figure 4C). To further dissect saffron’s effects, we assessed its effect on the relative abundance of bacterial groups at the order level (Figure 4D). This analysis showed differences at the order level, with some showing statistical significance. Indeed, bacteria from the Erysopelotrichales order were significantly more abundant in saffron-treated mice at day 10 when compared to DSS + vehicle-treated mice at day 10. Bacteria of the Enterobacteriales and Verrucomicrobiales orders were less abundant in saffron-treated mice at day 10. We also analyzed the relative abundance of significantly altered mucosa-associated bacterial groups at the genus level. Two groups were significantly less abundant, namely Mollicutes_RF39 and Flavonifractor, while Peptostreptococcaceae bacteria were more abundant in saffron-treated mice at day 10 (Figure 4E). There was only one unspecified Peptostreptococcaceae genus out of seven that was upregulated in saffron-treated mice. However, the overall abundance of Peptostreptococcaceae increased in DSS and DSS SFE mice from 0 to 2773.5 and 1446.25, respectively, very likely because of the effect of DSS. It is worth noting that the increase in overall Peptostreptococcacea genera was more pronounced in the DSS samples, highlighting a potential countereffect in saffron-treated mice that displayed a half-level increase in abundance.

### 3.3. Saffron Treatment Alters Primary Metabolites

**Figure 5:**
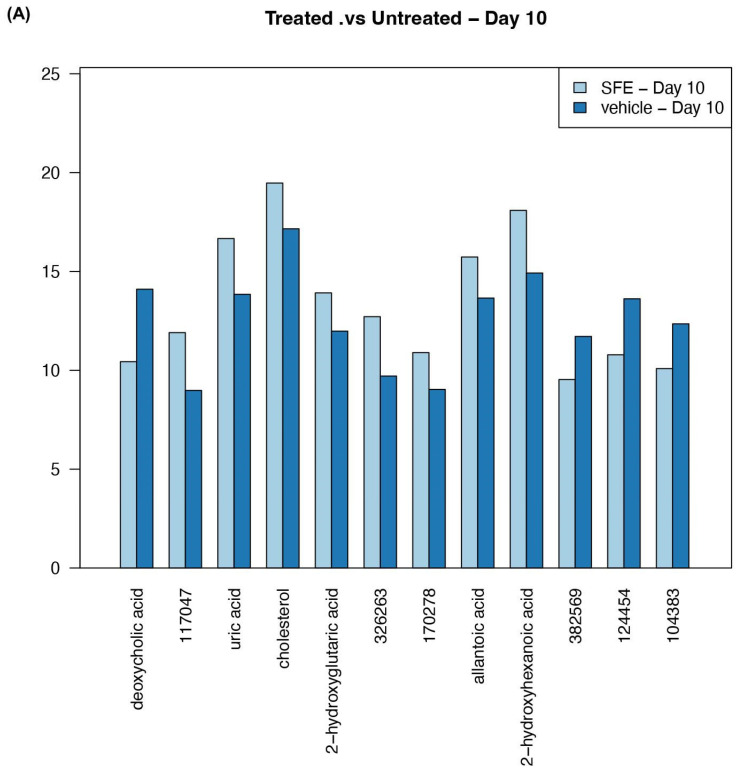

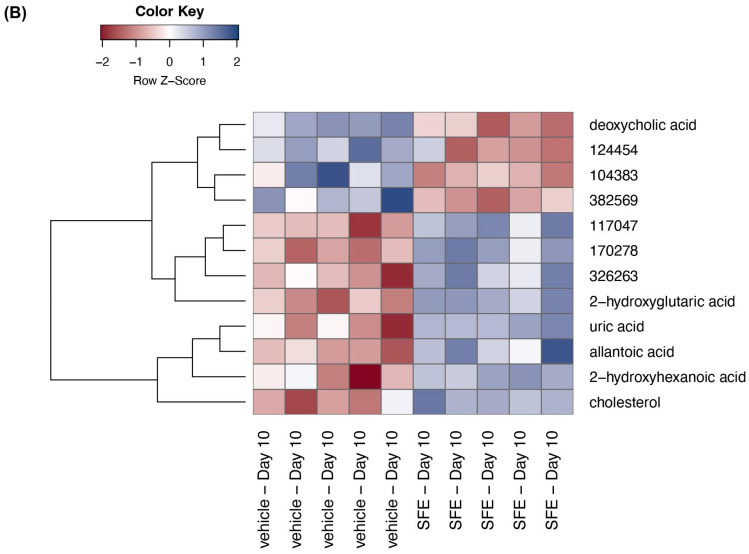
Untargeted primary metabolite analysis of stool samples collected from mice treated with DSS colitis at day 10. (**A**) Bar graph representing abundance of metabolites. (**B**) Hierarchy clustering of samples based on differential metabolites.

Untargeted analysis was performed on the stool pellets collected from DSS-treated mice at day 0 and day 10 with or without saffron intervention. We found that several metabolites were significantly altered in the stool of DSS-treated mice when comparing the stool of saffron-treated vs. untreated mice at day 0 and day 10 (Table S1). We compared stool metabolites between DSS + vehicle group and DSS + SFE group (n = 4–5 each) at day 10 (Table 1). Deoxycholic acid (DCA) increased with DSS treatment at day 10; saffron intervention reduced this DSS-induced upregulation (*p* < 0.001, FDR = 0.15). Some of the differentially identified metabolites included uric acid (*p* < 0.001, FDR = 0.039), cholesterol (*p* < 0.001, FDR = 0.039), 2 hydroxyglutaric acid (*p* < 0.001, FDR = 0.039), allantoic acid (*p* < 0.001, FDR = 0.044), and 2 hydroxyhexanoic acid (*p* = 0.001, FDR = 0.044), all of which were significantly increased in DSS-treated mice with saffron intervention at day 10. (Figure 5A,B). At day 10, there were other metabolites that were found to be increased with saffron treatment, such as β-sitosterol (*p* < 0.01, FDR = 0.074), 3 phenyl lactic acid (*p* < 0.01, FDR = 0.061), and 2-aminobutyric acid (*p* < 0.01, FDR = 0.075), although these metabolites did not cross the false discovery rate (FDR).

## 4. Discussion

In this study, we explored the effect of saffron, a common dietary additive, on microbial and metabolite composition in an experimental model of murine colitis. Saffron has been reported to be immunomodulatory [26]. Saffron contains active compounds, such as crocin and crocetin, which possess antioxidant properties [24,37,38]. These compounds can scavenge free radicals and reduce oxidative stress, protecting cells from damage and inflammation [39,40,41]. Saffron has been shown to possess anti-inflammatory properties by inhibiting the production of inflammatory mediators, such as cytokines and prostaglandins [42,43,44]. This modulation of the immune response can contribute to its therapeutic potential in conditions characterized by inflammation [26,42]. Saffron has been investigated for its neuroprotective effects, particularly in neurodegenerative diseases such as Alzheimer’s and Parkinson’s diseases [45,46,47,48]. It may help in preventing neuronal damage through antioxidant and anti-inflammatory mechanisms, promoting cell survival and enhancing neuroplasticity [48,49,50]. Saffron compounds have been reported to influence neurotransmitter systems, including serotonin, dopamine, and glutamate. These effects on neurotransmission may contribute to its potential in mood disorders and cognitive function [49,50,51]. Emerging evidence suggests that saffron may have epigenetic effects, meaning it could potentially influence gene expression patterns without altering the underlying DNA sequence [52,53,54]. These epigenetic changes might be involved in saffron’s therapeutic actions, although the specific mechanisms are not yet well understood. Here, we analyzed these anti-inflammatory-associated gut microbiota and metabolite changes. We found that saffron induced a richness in the mice microbiota, allowing OTUs to generally be detected at fewer sequencing reads when compared to samples collected at day 0 with DSS + vehicle and with DSS + SFE. This applied to DSS day 10 samples as well. Notably, in all four analyzed experimental sample sets, the rarefaction curves were saturated, showing that the depth of sequencing was adequate to account for most OTUs. The PCoA further highlighted saffron’s effect on individual mice in each group. 

The effects of saffron were first assessed at the bacterial phylum level, where the two prominent gut microbiota phyla in humans and mice are Firmicutes and Bacteroides. A stable ratio of these two phyla is a sign of a homeostatic gut microbiota [55]. DSS-treated mice had an increase in this ratio from day 0 to day 10, which might be due to an increase in Firmicutes and/or depletion of Bacteroides because of this colitis-inducing agent. Firmicutes were reported to be more prevalent in obesity and inflammation environments. However, this shift was not noticed in the saffron-treated mice, which displayed similar a Firmicutes/Bacteroides ratio at day 0 and day 10. This finding is very interesting as the richness noted in the rarefaction analysis does not seem to be random but more likely directed to counter the dysbiotic effect of DSS. 

At the order level, many bacterial orders showed clear differences between DSS-treated vs. DSS SFE-treated mice. Three orders were significant, with bacteria from the Erysopelotrichales order being significantly more abundant in saffron-treated mice at day 10 when compared to DSS-only-treated mice at day 10, while bacteria of the Enterobacteriales and Verrucomicrobiales orders were less abundant in saffron-treated mice at day 10. Schwab et al. have previously reported an increase in the low abundant Enterobacteriales, Deferribacterales, Verrucomicrobiales, and Erysipelotrichales in DSS-induced colitis mice [56]. Three of these orders were significantly reduced in abundance in the presence of saffron, which highlights saffron’s microbiota-modulating capacity to counter the dysbiotic effect of DSS. Only Erysopelotrichales showed a higher abundance among saffron-treated mice when compared to DSS-only-treated mice at day 10.

Dirk et al. showed that Crohn’s disease status correlated strongly with a decreased abundance of Erysipelotrichales [57], and this finding is consistent with the results reported in the present study, which show the protective effect of saffron.

Proteobacteria are often found to increase in number in IBD [57]. At the genus level, the anti-inflammatory effect of saffron through a modulation of the gut microbiota was further confirmed. Indeed, two genera showed significant decreases in abundance in saffron-treated mice, namely Flavonifractor and Mollicutes. These two genera have previously been shown to be associated with strong pro-inflammatory responses [58,59]. The known downregulation of these two well-established inflammatory bacteria supports the immune response modulation, as described in this study. However, the Peptostreptococcaceae genus was found to be more abundant in saffron-treated mice (at Day 10 vs. Day 0), and this is reportedly associated with colitis in DSS mouse models [60]. This finding led us to further explore all detected Peptostreptococcacaea in our samples, which were represented by seven genera that were cumulatively shown to be more abundant in DSS-treated mice than in DSS SFE mice. This finding on its own highlights the anti-colitis effect of saffron since, even in the presence of DSS, the increase in bacteria from these genera was slowed and kept at half the abundance obtained with DSS only. Indeed, the overall abundance of Peptostreptococcaceae increased in DSS and DSS SFE mice from 0 to 2773.5 and 1446.25, respectively, very likely due to the effect of DSS being partially countered by saffron treatment. In sum, we can state that saffron treatment at the genus level led to significant reductions in the abundance of three pro-inflammatory bacterial genera. 

These changes in bacterial composition were in parallel with changes in stool metabolite profiles. DCA was shown to be reduced in the saffron-treated mice. While DCA has been shown to have both anti- or pro-inflammatory effects depending on the model being investigated, some potentially deleterious effects may be revealed in DSS colitis [61,62]. It has been reported that DCA triggers NLRP3 inflammasome activation and aggravates DSS-induced colitis in mice [63], and high fat diets increase the level of fecal DCA, which contributes to colonic inflammation [64]. This may suggest a possible mechanism by which saffron exerts its protective effects on the gut. Other identified metabolites, such as uric acid, cholesterol, 2-hydroxyglutaric acid, allantoic acid, 2-hydroxyhexanoic acid, were significantly increased in DSS-treated mice with saffron intervention at day 10. The increase in uric acid in DSS-treated mice with saffron intervention may also play a role in mediating its therapeutic effect. It has been reported that uric acid is the predominant anti-oxidant molecule in plasma and is necessary and sufficient for the induction of type 2 immune responses [65]. Linoleic acid has been shown to be able to protect necrotic and apoptotic cell death induced by palmitic acid [66]. We found the upregulation of 3-phenyllactic acid in DSS + SFE group at day 10. The natural antimicrobial compound 3-phenyllactic acid exerts immune-modulatory effects [67]. In our metabolic analysis, we found increased levels of β-sitosterol in saffron-treated mice. In an experimental murine colitis model, it was reported that phytosterols exert anti-inflammatory effects [68]. β-sitosterol, a phytosterol, significantly inhibited colon shortening, lowered fecal hemoglobin contents, and reduced the severity of colitis in the middle and distal colon [69]. Gamma-aminobutyric acid (GABA) reduces pro-inflammatory cytokine production in the DSS-induced colitis colon [70] and, with saffron treatment, we found increased levels of 2-aminobutyric acid at day 10. These major shifts in metabolite level with saffron treatment might be related to the inflammatory protection mechanism of saffron in mice. It is important to note that, while some mechanisms have been proposed based on experimental studies and some clinical observations, the precise ways in which saffron exerts its therapeutic effects are still being investigated.

In conclusion, our study reports saffron’s modulating effects on the microbiome and metabolome, which reduced the inflammatory effects of DSS. These changes in the microbiome and metabolome are likely major contributors that lead to an anti-inflammatory immune response in DSS mice colitis models, as we have previously shown [26]. Further research is necessary to gain a comprehensive understanding of the underlying mechanisms and validate the therapeutic potential of saffron in different contexts.

## Figures and Tables

**Table 1:** Comparison of stool metabolites between DSS + vehicle group and DSS + SFE group (n = 4–5 each) at day 10.

BinBase Name	Treated vs. Untreated Day 10 Log2-Fold-Change	Treated vs. Untreated Day 10 *p*-Value	Treated vs. Untreated Day 10 FDR
deoxycholic acid	−3.664	<0.0001	0.015
2-hydroxyglutaric acid	1.934	<0.0001	0.039
cholesterol	2.314	<0.0001	0.039
uric acid	2.822	<0.0001	0.039
2-hydroxyhexanoic acid	3.168	0.001	0.044
allantoic acid	2.077	0.001	0.044
linoleic acid	2.060	0.001	0.050
3-phenyllactic acid	2.016	0.002	0.061
beta-sitosterol	2.039	0.003	0.074
2-aminobutyric acid	1.963	0.003	0.075
melibiose	−2.559	0.003	0.076
urocanic acid	2.248	0.004	0.076
aconitic acid	2.193	0.004	0.081
N-acetylglycine	−2.132	0.006	0.105
N-acetylornithine	1.191	0.007	0.108
fructose	−3.187	0.009	0.116
homovanillic acid	2.411	0.008	0.116
malonic acid	1.719	0.007	0.116
mannose	−1.596	0.009	0.116
mevalonic acid	1.537	0.008	0.116
N-acetylglutamate	1.123	0.009	0.121
citramalic acid	2.546	0.010	0.126
phytol	1.454	0.010	0.126
3-aminoisobutyric acid	1.689	0.015	0.145
arachidonic acid	1.531	0.013	0.145
beta-glutamic acid	1.792	0.013	0.145
gluconic acid	2.386	0.014	0.145
lactitol	−1.326	0.015	0.145
N-acetyl-D-mannosamine	−1.818	0.015	0.145
pseudo uridine	1.656	0.015	0.145
thymine	1.995	0.015	0.145
sucrose	−1.998	0.016	0.146
2-deoxypentitol	1.011	0.018	0.146
tyrosine	−1.158	0.018	0.146
4-hydroxyphenylacetic acid	−2.761	0.019	0.147
beta-alanine	1.313	0.020	0.151
glucose	−1.772	0.020	0.151
orotic acid	1.444	0.022	0.155
1-kestose	−2.516	0.024	0.165
quinolinic acid	1.073	0.025	0.170
galactitol	−1.096	0.028	0.172
N-acetyl-D-galactosamine	−2.163	0.027	0.172
squalene	1.114	0.029	0.179
cytosine	1.161	0.032	0.189
3-hydroxy-3-methylglutaric acid	0.861	0.033	0.190
glutaric acid	−1.407	0.034	0.191
3,6-anhydro-D-galactose	−0.833	0.038	0.204
xylulose	0.898	0.037	0.204
4-hydroxybutyric acid	1.196	0.043	0.222
dihydrocholesterol	1.292	0.045	0.227
epsilon-caprolactam	−1.141	0.045	0.227
cis-gondoic acid	1.116	0.049	0.230
pipecolinic acid	1.087	0.049	0.230
raffinose	−3.357	0.048	0.230

All the unidentified metabolites were excluded in this table.

## Data Availability

Data for this manuscript can be obtained by contacting the corresponding authors.

## Supplementary Materials

The following supporting information can be downloaded at: https://www.mdpi.com/article/10.3390/cimb45070351/s1, Table S1. Stool metabolite comparison in DSS + SFE group (n = 4–5 each) at two time points (day 0 and day 10).
