# Supplementary material for: Microbiomic and Metabolomic Analyses Unveil the Protective Effect of Saffron in a Mouse Colitis Model
Source: Curr Issues Mol Biol. 2023 Jun 30;45(7):5558–74. doi: 10.3390/cimb45070351 (PMC10378474; doi:10.3390/cimb45070351)
Supplement: Supplementary file 1 [file cimb-45-00351-s001.zip › cimb-2423294-supplementary.pdf]

Supplementary information

Table S1: Stool metabolite comparison in DSS+SFE group (n=5 each) at two time points (day 0 and day 10).

BinBase name	DSS+SFE 10 vs DSS+SFE 0 Log2-Fold-change	Treated day 10 vs day 0 P-value	Treated day 10 vs day 0 FDR
1-monostearin	2.015	0.010	0.028
1,5-anhydroglucitol	3.320	0.000	0.001
2-aminobutyric acid	2.821	0.000	0.000
2-deoxypentitol	2.062	0.000	0.001
2-deoxytetronic acid	1.016	0.023	0.055
2-hydroxybutanoic acid	4.861	0.000	0.000
2-hydroxyglutaric acid	0.819	0.022	0.053
2-hydroxyhexanoic acid	4.944	0.000	0.000
2-monoolein	−3.887	0.002	0.007
2'-deoxyguanosine	−2.183	0.006	0.020
3-aminoisobutyric acid	1.364	0.007	0.021
3-epicholic acid	−2.052	0.007	0.022
3-hydroxy-3-methylglutaric acid	2.975	0.000	0.000
3-hydroxybutyric acid	2.810	0.000	0.000
3-phenyllactic acid	3.883	0.000	0.000
3,4-dihydroxyphenylacetic acid	−2.410	0.001	0.003
4-aminobutyric acid	2.848	0.018	0.046
4-hydroxybenzoic acid	1.515	0.005	0.015
4-hydroxybutyric acid	3.576	0.000	0.000
4-pyridoxic acid	−1.051	0.011	0.031
5-aminovaleric acid	1.880	0.001	0.006
5-methoxytryptamine	−1.652	0.009	0.027
aconitic acid	1.609	0.017	0.044
aminomalonate	1.897	0.009	0.026
arachidonic acid	3.246	0.000	0.001
beta-gentiobiose	2.197	0.005	0.018
cellobiose	−1.796	0.011	0.032
chenodeoxycholic acid	−1.199	0.008	0.024
cholesterol	3.397	0.000	0.000
citramalic acid	3.876	0.000	0.002
conduritol-beta-epoxide	4.549	0.000	0.000
creatinine	2.181	0.000	0.001
cysteine	1.565	0.006	0.020
cystine	3.832	0.000	0.000
daidzein	2.264	0.000	0.003
deoxycholic acid	−7.352	0.000	0.000
dihydro-3-coumaric acid	−4.553	0.000	0.000
docosahexaenoic acid	1.732	0.004	0.015
erythritol	1.655	0.003	0.012
ferulic acid	−1.790	0.004	0.014
fumaric acid	1.939	0.001	0.004
galactinol	1.559	0.002	0.010
gluconic acid	3.454	0.000	0.002
glucose	−1.894	0.003	0.011

glutamine	−1.520	0.017	0.044
glycerol-3-galactoside	−1.152	0.019	0.048
glycine	1.210	0.003	0.011
glycolic acid	2.514	0.000	0.000
glycyl tyrosine	−1.408	0.004	0.013
guanine	−5.746	0.000	0.000
hydrocinnamic acid	−1.871	0.004	0.014
hypoxanthine	−4.625	0.000	0.001
isothreonic acid	−1.384	0.018	0.046
L-DOPA	2.202	0.000	0.001
lactitol	−3.038	0.000	0.000
levoglucosan	1.021	0.008	0.025
lithocholic acid	−2.028	0.000	0.003
maleimide	1.002	0.014	0.039
malic acid	3.090	0.000	0.000
malonic acid	3.535	0.000	0.000
myo-inositol	2.821	0.000	0.000
N-acetylglutamate	−1.182	0.007	0.023
nicotianamine	2.723	0.000	0.001
O-acetylserine	1.253	0.011	0.032
oleic acid	−1.947	0.003	0.010
oxamic acid	2.533	0.000	0.000
palmitoleic acid	1.820	0.002	0.007
parabanic acid	2.505	0.001	0.004
pentitol	2.016	0.000	0.002
pentose	−2.381	0.002	0.008
phytol	−1.587	0.012	0.032
phytosphingosine	2.109	0.008	0.023
pinitol	4.600	0.000	0.001
piperidone	1.594	0.001	0.003
quinic acid	1.991	0.000	0.003
quinolinic acid	1.530	0.003	0.012
raffinose	1.848	0.018	0.046
ribose	−1.936	0.007	0.023
saccharic acid	2.407	0.000	0.001
saccharopine	−1.474	0.002	0.007
sarcosine	1.130	0.008	0.025
sophorose	−2.059	0.016	0.042
squalene	1.139	0.008	0.023
tagatose	1.258	0.006	0.020
tocopherol alpha-	−2.128	0.004	0.015
tyrosine	−1.646	0.001	0.005
uric acid	4.088	0.000	0.000
urocanic acid	1.057	0.006	0.020
vanillic acid	2.460	0.000	0.001
xanthosine	2.255	0.000	0.003
xylose	−2.302	0.001	0.003
zymosterol	1.504	0.002	0.009

*All the unidentified metabolites were excluded in this table.
At day 0 these are baseline values but at day 10 the alterations of metabolites are due to effect of DSS+SFE20mg (combine effect of DSS+SFE20mg)

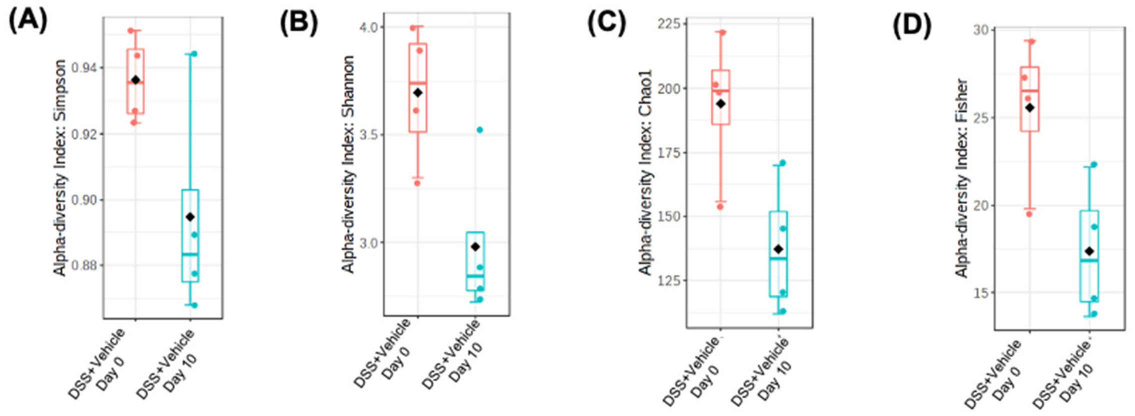

Supplementary Figure S1: Microbial diversity comparison between two timepoints day 0 and day 10 for DSS+Vehicle group. The OTU number representing the bacterial species richness of the microbiota was estimated for alpha diversity using the, A) Simpson (p value:0.087) , B) Shannon index (p value:0.025), C) Chao1 (p value:0.024), D) Fischer (p value: 0.027).

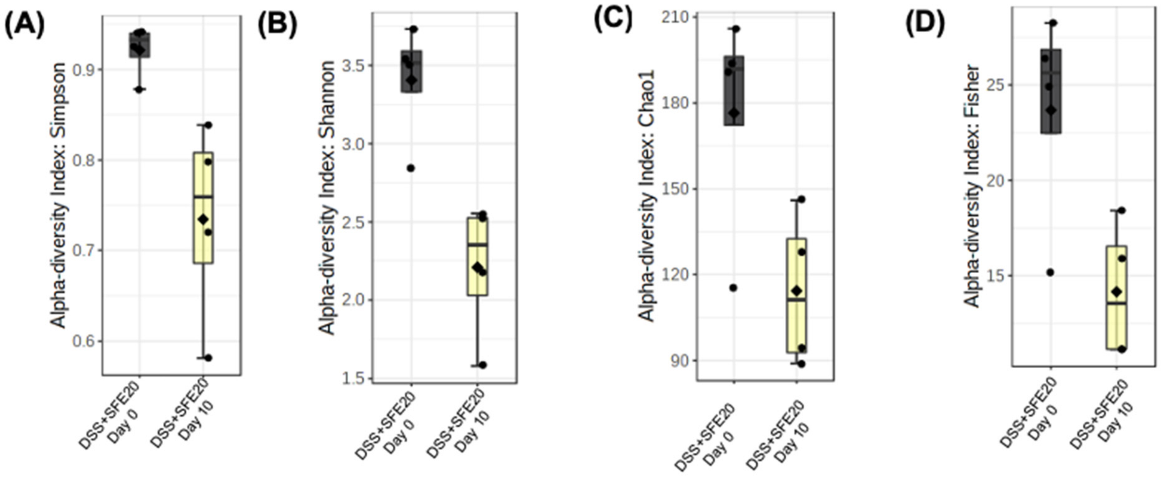

Supplementary Figure S2: Microbial diversity comparison between two timepoints day 0 and day 10 for DSS+SFE20 group. The OTU number representing the bacterial species richness of the microbiota was estimated for alpha diversity using the , A) Simpson (p value:0.041) , B) Shannon index (p value:0.007), C) Chao1 (p value:0.050), D) Fischer (p value: 0.039).
